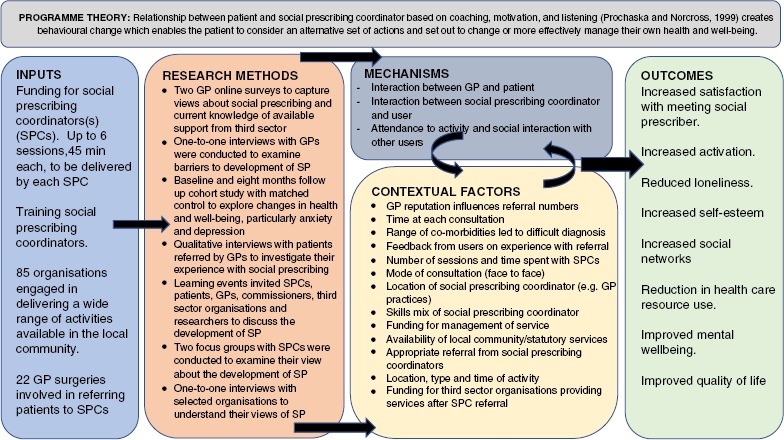# A realist evaluation of social prescribing: an exploration into the context and mechanisms underpinning a pathway linking primary care with the voluntary sector

**DOI:** 10.1017/S1463423617000706

**Published:** 2017-12-07

**Authors:** Marcello Bertotti, Caroline Frostick, Patrick Hutt, Ratna Sohanpal, Dawn Carnes

**Affiliations:** 1 Senior Research Fellow, Institute for Health and Human Development, University of East London, Water Lane, Stratford, London, UK; 2 Research Fellow, Institute for Health and Human Development, University of East London, Water Lane, Stratford, London, UK; 3 Clinical Associate, UCL Department of Primary Care and Population Health; 4 Post-Doctoral Researcher, Barts and The London School of Medicine and Dentistry, Centre for Primary Care and Public Health, London, UK; 5 Barts and The London School of Medicine and Dentistry, Centre for Primary Care and Public Health, Queen Mary University of London, London, UK

**Keywords:** community health, health inequalities, link workers, primary care, public health, realist evaluation, social prescribing

## Abstract

This article adopts a realist approach to evaluate a social prescribing pilot in the areas of Hackney and City in London (United Kingdom). It unpacks the contextual factors and mechanisms that influenced the development of this pilot for the benefits of GPs, commissioners and practitioners, and reflects on the realist approach to evaluation as a tool for the evaluation of health interventions. Primary care faces considerable challenges including the increase in long-term conditions, GP consultation rates, and widening health inequalities. With its emphasis on linking primary care to non-clinical community services via a social prescribing coordinator (SPC), some models of social prescribing could contribute to reduce the burden on primary care, tackle health inequalities and encourage people to make greater use of non-clinical forms of support. This realist analysis was based on qualitative interviews with users, commissioners, a GP survey, focus groups and learning events to explore stakeholders’ experience. To enable a detailed analysis, we adapted the realist approach by subdividing the social prescribing pathway into stages, each with contextual factors, mechanisms and outcomes. SPCs were pivotal to the effective functioning of the social prescribing service and responsible for the activation and initial beneficial impact on users. Although social prescribing shows significant potential for the benefit of patients and primary care, several challenges need to be considered and overcome, including ‘buy in’ from some GPs, branding, and funding for the third sector in a context where social care cuts are severely affecting the delivery of health care. With its emphasis on context and mechanisms, the realist evaluation approach is useful in understanding how to identify and improve health interventions, and analyse in greater detail the contribution of different stakeholders. As the SPC is central to social prescribing, more needs to be done to understand their role conceptually and practically.

## Introduction

Primary care in the United Kingdom currently faces a number of key challenges including:

(i) About 20% of people attend GP surgeries for problems that are primarily social rather than medical (Torjesen, 2016).
(ii) A rising tide of long-term conditions which is set to grow by 5 million in the next 10 years (DoH, 2013).
(iii) Growing health inequalities which result in long-term medical conditions disproportionately affecting people in deprived areas (Hutt and Gilmour, 2010; Marmot *et al*., 2010; Cawston, 2011).

In an attempt to seek solutions to these problems, the concept of social prescribing holds significant promise (South *et al*., 2008). The Social Prescribing Network (SPN) defines social prescribing as ‘a means of enabling GPs and other frontline healthcare professionals to refer patients to a link worker – to provide them with a face-to-face conversation during which they can learn about the possibilities and design their own personalised solutions, i.e. “co-produce” their “social prescription” – so that people with social, emotional or practical needs are empowered to find solutions which will improve their health and well-being, often using services provided by the voluntary and community sector’ (SPN, 2016: 19).

A range of different models of social prescribing have emerged in the last 10 years. A useful way to conceptualise these is Kimberlee (2015) who has arranged models into signposting, light, medium and holistic, according to a range of aspects and, crucially, the level of support provided by link workers to patients. The research analysed in this article is based upon the ‘holistic’ model which includes a clear referral pathway, an holistic view of patient’s needs and aspirations, and an intense level of support provided by the link worker.

The NHS Five Year Forward View (NHS, 2014) recognised social prescribing as an important model for the future of the NHS. Moreover, the General Practice Forward View (NHS, 2016) recognised social prescribing as one of the 10 high impact interventions to release capacity in GP surgeries. Capacity would be released by making greater use of the third sector as an asset available in the community (Morgan and Ziglio, 2010).

Some reviews of social prescribing are now available (CRD, 2015; Thomson *et al*., 2015; Ward, 2016; Bickerdike *et al*., 2017). They examine the context, outcomes and impact of social prescribing on a vast array of health and social outcomes in an attempt to show its effectiveness (Dayson *et al*., 2013; Carnes *et al*., 2015) and cost-effectiveness (Grant *et al*., 2000; Kimberlee, 2016). As highlighted in a recent systematic review (Bickerdike *et al*., 2017), much less is known about the challenges of implementing social prescribing in practice. Yet, an appropriate discussion of implementation challenges is a crucial part of policy development as these have immediate implications for outcomes and long-term sustainability. Moreover, an evaluation discussing implementation challenges would be particularly useful to aid commissioners, and practitioners in their effort to ‘learn’ from prior experience and design more effective health interventions.

In exploring implementation challenges, this article aims to address the following question: what worked in the social prescribing pilot in City and Hackney, for whom and under what circumstances? This discussion may provide a road map for future social prescribing pilots and inform further development of social prescribing models across the United Kingdom and beyond. In this context, the realist approach appears to be an appropriate methodology to explore implementation challenges and is particularly useful in considering the influence of contextual factors in intervention development and also in identifying the key mechanisms that make an intervention work. The realist evaluation approach is centred upon the notion that the analysis of ‘what works’ in evaluation is not sufficient in securing the improvement of evaluation science (Pawson and Tilley, 1997). Attention should also be paid to the analysis of ‘what works, for whom and under what circumstances’.

The realist approach has gained considerable ground in the last few years and work is underway to ensure that this approach becomes an established part of evaluative practice within the Medical Research Council (Fletcher *et al*., 2016) and more widely (Raines *et al*., 2016). It has been mostly used in the evaluation of health systems (Marchal *et al*., 2012), illicit drug deterrence programmes (Leone, 2008), shared care in mental health (Byng *et al*., 2008), community-based participatory research (Jagosh *et al*., 2015), and modernisation of health services (Greenhalgh *et al*., 2009). In relation to social prescribing more specifically, we could only find one realist evaluation (Arain, 2015).

Through the realist approach to evaluation lens, this article explores the specific experience of social prescribing in City and Hackney, one of the largest pilots in the United Kingdom. This article differs from other evaluations reported from the same pilot which have focussed on assessing health outcomes and process from a different perspective. (Bertotti *et al*., 2015; Carnes *et al*., 2015). During the period between February 2014 and July 2015, 23 GP surgeries located in the London Borough of Hackney and the City of London referred 737 patients with symptoms of social isolation, mild-moderate mental health problems, presenting with a social problem, or frequent attenders to GP/A&E to three social prescribing coordinators (SPCs). SPCs met each patient for up to six, 40 min long, sessions to co-produce a well-being plan resulting from discussions about the needs and aspirations of each patient and the availability of local support services. This led to the referral of patients to a total of 85 community organisations in the borough which delivered physical activity classes, health advice, networking activities (eg, lunch clubs), psychological support, art and other services. SPCs were employed by a local voluntary organisation which managed the development of social prescribing supported by funding from the City and Hackney Clinical Commissioning Group. The objectives of the social prescribing pilot in City and Hackney included (City and Hackney CCG, 2013):

∙ Enable individuals to feel more in control and improve health and well-being
∙ Reduce social isolation
∙ Increase GP awareness of what is happening in the community and vice versa
∙ Reduce GP visits and A&E attendance

## Methodology and methods: realist evaluation

This section introduces the key concepts underpinning the realist approach to evaluation and discusses its application to the specific case of City and Hackney.

The realist approach assumes that programmes are ‘theories incarnate’ (Greenhalgh *et al*., 2016:2). The article considers an overall hypothesis, contextual factors and an overarching mechanism. The initial hypothesis was that ‘a social prescribing intervention improves wellbeing outcomes for patients suffering from isolation and mild mental health problems by providing a support mechanism (GPs, social prescribing coordinators and community organisations) which enable each patient to consider a set of alternative actions and thus embark on changing or more effectively managing their current health’. Social cognitive theory is one of the chosen conceptual models that underpins the process of behavioural change (Bandura, 1986). In social prescribing, behavioural change leads to improved mental and physical well-being in three key ways: first, the combined effect of one-to-one interaction between the patient and SPC in the form of coaching, motivation and listening (Prochaska and Norcross, 2009); second, the social interaction between the patient and the group of people involved in running community activities; and third, the social interaction within other community activities. Through the support received from SPCs and social interaction in the community, patients move through different stages, ultimately finding themselves empowered to change their own circumstances (Hibbard and Gilburt, 2014).

Theories are then tested by analysing the Context, Mechanisms and Outcome (CMO) configuration which is based on the notion that the Outcome of interest is generated by the interaction between Context and Mechanism. The analysis of the interaction between Context and Mechanism assists in the testing and development of ‘middle range’ theories about the functioning of the programme. Context consists of a range of factors that may influence the ability of mechanisms to produce outcome changes. These may include the cultural, historical, and policy background in which the intervention is being implemented (Pawson and Tilley, 2004). The identification of important contextual influences on mechanisms pose a considerable challenge in complex interventions which are multi-components (Moore *et al*., 2015). Pawson and Tilley (2004) argue that ‘mechanisms describe what it is about programmes and interventions that bring about any effects’ and ‘mechanism refers to the ways in which any one of the components [of the intervention] or any set off them, or any step or series of steps brings about change’ (p. 6).

In using the realist review and CMO configuration to understand, test and refine the initial middle range theories, we defined the specific model of social prescribing in City and Hackney as the intervention under examination, and identified the interaction between patient and GPs, patient and SPCs, and patient and community/statutory organisations as the main components underpinning the intervention. However, as described below, the formulation of hypothesis, context, mechanisms and outcomes for each of the components of the intervention provides us with specific insights that lead to a more complete understanding of what works, for whom and under what circumstances (Table 1).

### Methods

In order to uncover the contextual factors and mechanisms underpinning social prescribing, we drew upon a number of methods including two quantitative GP online surveys with GP surgeries in Hackney, qualitative interviews with stakeholders (17 patients using the social prescribing services, three community organisations, focus groups and individual interviews with three SPCs, two interviews with commissioners, two interviews with GPs), two learning events involving a wide range of stakeholders including SPCs, commissioners, community organisation representatives, and service users, and observations of sessions between SPCs and individuals (Table 2). About 85 voluntary sector organisations received referrals from SPCs. These included lunch clubs, walking groups, psychological counselling, gardening, and bereavement support amongst others. Primary data collection and analysis was also supported by monitoring data provided by SPCs as part of the evaluation. These included data on the number of people being referred at each stage of the pathway (GP, SPCs and community organisations), information on the type and number of community organisations involved in the referral process, and number and length of consultations with patients. This information was used to provide a picture of the pathway and to analyse potential opportunities and challenges faced by patients referred.

### Sampling

from the total sample of service users referred by their GPs, we attempted to select a random sample in order to provide a more objective view of their experience rather than relying on SPCs to select participants on our behalf. However, we were only able to select and interview seven with this method as the number consenting through this strategy was small. The remainder (10) were selected with the help of SPCs who contacted service users and asked if researchers could contact them for an interview. Following consent, researchers contacted service users and sought informed consent (see below for details). In terms of the other methods used in the evaluation, both GP online surveys were sent to all the GP practices in borough.

### Data collection

Most qualitative data was collected between January and June 2015. Data collection was primarily face-to-face, but in two cases telephone interviews were conducted. NHS ethics was obtained from NRES East Midlands (14/EM/1076). Informed consent was secured at the beginning of each interview and digitally recorded. Researchers provided information about the aims of the research, explained the reasons for the interview, and sought informed consent by asking respondents to sign a consent form. Participants were asked open-ended questions about their experience with GPs, SPCs, and voluntary organisations. GP online surveys were conducted through Survey Monkey, administered through the Clinical Commissioning Group and sent to all GP surgeries in Hackney.

### Analysis

Evidence generated through the qualitative work was analysed thematically, discussed amongst researchers across the two academic institutions to ensure inter-rater reliability, and triangulated with other sources of information to increase internal validity. GP online surveys were analysed descriptively using Statistical Package for Social Sciences.

## Findings

The overall hypothesis underpinning the realist evaluation of social prescribing in City and Hackney is that social prescribing improves well-being outcomes for patients suffering from isolation, and mild mental health problems. It provides a mechanism of support that enables each individual participant to consider a set of actions they may be willing to take, and thus embark on the journey to socially re-activate themselves, change their behaviour and, ultimately, their health.

Our data collection shows that beneficial outcomes for patients result from the combination of multiple stages working together effectively. The realist evaluation approach enabled us to identify these three stages as the interaction between the patient and three other stakeholders: the GP (stage one), the SPC (stage two), and community organisations (stage three). We discuss hypotheses, contexts, and mechanisms for each of these three stages below (Table 1).

### Stage one: GP referral process

The first stage of the social prescribing realist evaluation model for City and Hackney is the GP referral process. The hypothesis for this stage is that many patients experiencing social isolation, mild-moderate mental health problems, social problems, or frequent attenders to GP/A&E would be referred to a SPC by their General Practitioners. Stakeholders involved in the development of social prescribing argued that GPs are in a powerful position to advocate a new approach to their patients as they have a consolidated reputation. Such reputation can ensure strong compliance from patients and improve attendance to SPC consultations. The mechanism identified to explain this stage of the process is the interaction between GP and patient resulting in a referral to a SPC which is considered as the outcome of the Context, Mechanism, Outcome (CMO) configuration.

The evaluation revealed a range of contextual factors influencing the number of referrals and their appropriateness. Before the beginning of the intervention, all GPs completing an online survey (*n*=52) recognised the value of social prescribing for improving well-being for patients, particularly socially isolated individuals. However, when asked their opinions about healthcare use, 50% of participants (*n*=25) responded ‘no’ to social prescribing being useful for reducing GP attendance, and 80% responded ‘no’ to social prescribing being useful for reducing A&E admissions (*n*=44). The same survey showed that GP knowledge of surrounding community organisations was poor overall with 61% knowing ‘a little or not at all’ about the presence of local community organisation that could offer support and identifying their lack of knowledge of what is available as one of the major obstacles to referral. Referrals of patients to community activities was seen as challenging because databases about community organisations were out of date and activities delivered by community organisations experience a high turnover.

The major contextual factors influencing this stage of the referral process included the pervasiveness of GP clinical training coupled with the need to assess and support patients in about 11.2 min consultation sessions (Curtis, 2014). This meant that in a pressurised environment a clinical diagnosis was sometimes preferred over other options such as social prescribing. As one GP interviewed reported:

‘*The terrible thing is that I referred five but I should have referred about 15 times that. Although I am very enthusiastic about it, it is hard to keep in front of your mind, and that’s the challenge*!’(GP)

Further evidence from qualitative interviews with participants and online surveys of GPs shows that more needs to be done to promote the opportunities and services offered by social prescribing amongst participants and GPs. Data from learning events and stakeholders’ interviews showed that clinicians would appreciate more information about patients they had referred to SPCs (Bertotti *et al*., 2015). Almost all patients interviewed in the qualitative study were not aware of the term ‘social prescribing’ but rather remembered the name of their SPCs.

‘*I have no idea who or what you are talking about, but sounds a good idea, I don’t know why I was referred…*’ (service user)

Although the online survey showed that all GPs felt social prescribing could help with their patient well-being, an additional contextual factor is that belief in the social prescribing intervention takes time, hence the need for persistent communication from SPCs to ‘remind’ GPs of the availability of the service. Most referrals to social prescribing took place in a few GP surgeries (six GP surgeries referred 50% of total referrals). Other strategies to maximise referrals from GP surgeries included the co-location of SPCs in GP surgeries and the use of promotional material within GP surgeries.

### Stage two: consultation with the SPC

The second stage of analysis is based on the hypothesis that face-to-face consultation between patient and SPC generates an opportunity for the patient to explore their needs and aspirations. As a result of one or more consultations, the SPC helps the patient in accessing relevant activities available from statutory and community sectors which could contribute to the ‘activation’ of the patient. The mechanism underpinning this stage of the intervention is the interaction between SPC and patient.

Data from qualitative interviews with participants and stakeholder interviews carried out as part of the evaluation of social prescribing in City and Hackney show that face-to-face sessions, and length of each consultation session with SPCs are important, particularly at the first meeting. This appears to build trust and gives the opportunity for patients to discuss their problems in a non-clinical context, for a period that exceeds what is normally offered by GPs or other professionals in a clinical setting.

‘*You feel able to offload if you need to, discuss your fears – it’s about not being so hard on myself and validating myself*.’ (service user)

The skills mix of the SPC are of considerable importance within the context of their sessions with patients. In City and Hackney, SPCs had an educational background in psychotherapy, psychology and coaching, and had previous experience working in the voluntary sector as well as considerable listening and empathetic skills.

The level of intervention offered by the SPC may vary considerably from straightforward signposting, requiring a detailed knowledge of local organisations available to the patient, to a more intensive coaching-style intervention for those patients needing to overcome barriers before moving on to the next step. In City and Hackney, 20% (*n*=99) of users attended at least two, 40 min, sessions with SPCs, primarily when the discussion between patient and SPC had not reached clarity over the choice of community activities and patients needed to explore their needs and aspirations further.

‘*For any significant behavioural change, you need a bit longer to work with somebody so it is much more than just signposting someone on to a community service, it’s spending that time with somebody to help them work out where they want to go next*.’ (SPC)

and one service user reported:

‘*She was just absolutely wonderful…she was just right…I told her what had happened and that seemed to get it out of my head a bit. All these years it’s just been in my head*.’ (service user)

However, not all respondents shared a positive experience with their SPCs. One respondent felt that the lack of eye contact during a session was an important problem potentially highlighting the need for more active listening and attention towards what the patient had to say. As the respondent argued:

‘*It would have been much nicer if they had had a conversation face-to-face cause it felt like I was sitting there and they were at the desk trying to write everything down quickly…I think a better way would be someone is giving you eye-contact rather than just writing things down and you’re thinking what are they writing?*’ (service user)

This response also highlights a wider point about the importance of face-to-face interaction as a basis for more active listening and greater communication between patient and SPC.

### Stage three: interaction with community/statutory organisations

The third stage of the intervention concerns the interaction between patients and local community/statutory organisations. The hypothesis is that patients engage with activity in the community thereby improving their well-being, social interaction and control over their health. The mechanism is the interaction between the patient, other participants involved in community support activities by support organisations, and the people employed by community organisations to run activities attended by social prescribing patients. Data shows that patients were referred to a total of 82 organisations, although about 10% of these received the vast majority of referrals. The need for a large number of support organisations was driven by a sophisticated demand coupled with the need to access activities at short commuting distance. Although monitoring data could not be collected, qualitative interviews demonstrate the benefits of referral to community organisations which led to tackling experiences of social isolation and/or loneliness.

‘*Last summer was the best one I’ve had since my surgery because I’ve got somewhere to go….. It’s better to sit with company sometimes than sit by yourself*.’ (service user)

‘*Best thing has been meeting new people and making friends. My mobile full up with names and numbers of friends before it was just family and doctor’s number*.’ (service user)

Some qualitative evidence suggests that this may depend upon the specific need and aspirations of each individual, with a calibrated mix of sessions with the SPC and community organisations as a positive way forward.

An additional important contextual aspect raised by interviews with stakeholders and at learning events was the lack of funding available for the delivery of community activities. Community organisations could offer some services to social prescribing participants but third sector respondents criticised the lack of a sustainable long-term funding provision. There was an expectation that the third sector had significant spare capacity and would be able to accommodate for all the referrals, regardless of their number. However, in their responses, representatives from the third sector drew attention to the ‘unprecedented’ level of funding cuts for social care as a major problem for continued and sustainable delivery of social prescribing services.

## Discussion

This study shows that ‘what works, for whom, in what circumstances’ can be best understood through the disaggregation of the intervention in discrete CMO configurations. Whilst it is possible to develop an overall CMO configuration (see Table 1), the analysis of social prescribing in City and Hackney is more insightful if its sub-components are considered. Mechanisms and contextual factors from each component need to operate as effectively as possible to produce the overall outcome of improving health and well-being for service users.

**Table 1:** Context, Mechanisms and Outcome (CMO) configuration for the social prescribing in City and Hackney (source: authors)

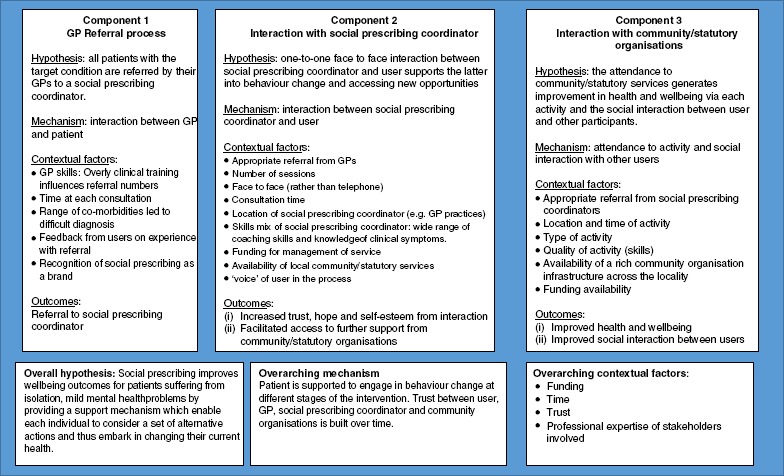

### What works

It is difficult to identify a key mechanism that is overarching and enables social prescribing to work as many components need to operate at different stages for the intervention to function effectively. However, the relationship between SPC and patient appear to be one of the main mechanisms that makes the intervention ‘what works’. Other evaluations (Friedli *et al*., 2012; Walker and Thirlwall, 2015; Innovation Unit, 2016) have highlighted the centrality of this role with an emphasis on patients’ choice, and qualitative evidence shows patients’ appreciation of this interaction. The ‘therapeutic alliance’, empathetic listening skills, genuineness and a non-judgemental approach are considered the core conditions needed to promote behaviour change in clients (Rogers, 1962). The more structured involvement of the SPC is likely to benefit the patient and support them in their journey towards activation and behaviour change. Our interviews show the crucial role of SPC style of ‘coaching’ which takes us firmly into the field of psychotherapy which, however, runs alongside a risk of ‘pathologising’ people. Referral to counselling interventions can be perceived as having a stigma attached, whereas social prescribing has a broader remit, with a focus on skills and integration into the wider community.

Thus, when patients are given agency and control over their time with non-imposing support from qualified SPCs who are empathetic and have a good knowledge of the social support infrastructure available locally, social prescribing is likely to have a beneficial impact on service users, particularly service users with multiple and complex needs.

The nature and level of interaction between patient and SPC depends upon the patient and their needs and aspirations. Some patients may not need in-depth support and can be easily ‘signposted’ to activities with little engagement from the SPCs. Yet, the evaluation of social prescribing in City and Hackney (Bertotti *et al*., 2015) has shown that about 61% of patients contacted required more than one consultation with their SPCs.

From a more conceptual point of view, when the relationship between SPC and patient develops successfully, the patient develops a strong sense of self-efficacy, feeling of control and a willingness to take on and persist with new and difficult tasks (Coulter and Ellins, 2006). The motivation and support offered by SPCs creates the basis for behaviour change such as greater willingness to participate in chosen community activities. Greater motivation has also been compared with the idea of activation (Hibbard and Gilburt, 2014) which in turn have shown that greater activation is associated with greater attendance to screenings and check-ups, and higher engagement in healthy behaviours like taking exercise (Tabrizi *et al*., 2010).

### For whom

The issue of targeting is an important part of the development of social prescribing which had an early focus on people with mild and moderate mental health problems (Friedli and Watson, 2004), but then became increasingly centred upon tackling long-term conditions with the main aim of improving patients’ self-care (DoH, 2006). Current models of social prescribing have expanded the target group to include support for people with employment (Steadman *et al.*, 2017), housing and debt-related issues (NHS, 2016).

In relation to City and Hackney, the target population included people experiencing mild and mental health problems and social isolation. A survey (*n*=183) carried out as part of the evaluation showed that on average participants were clinically anxious and borderline depressed. Qualitative interviews also revealed that social prescribing users are likely to suffer from a range of co-morbidities and be socially isolated. The intervention targeted an older age group (the median was 56), three out of five people lived alone, only 7% were in employment (30% retired). They therefore experienced health inequalities and belong to the intended target population. Qualitative interviews with participants and SPCs emphasised the importance of increased self-esteem, and a renewed sense of purpose as an outcome from their interaction and, ultimately, into taking action concerning their own health and well-being. Thus, social prescribing seems to work for all those patients who need support and motivation to act upon improving their own health and well-being, particularly if their needs are non-clinical or have a non-clinical component.

However, it is important to carefully consider some trends taking place in Hackney, also potentially occurring in other parts of the country where social prescribing is being implemented. In Hackney, there has been a growing emphasis toward an increasing number of referrals to show value for money. This has lead the steering group to consider self-referrals in addition to referrals from GP practices. Whilst on the one hand self-referrals open up the opportunity for more people to be supported by social prescribing, it also means that the work of SPCs will be spread more thinly and likely to encounter a number of people who would normally be able to identify community activities with little help, independently from support by SPCs. This would effectively open up the programme to a lack of specific targeting towards vulnerable groups which could lead to a waste of precious and ever scarcer resources.

### Under what circumstances

The circumstances under which the intervention can lead to the expected outcomes depends upon the analysis of contextual factors. Contextual factors include a vast array of influencing variables including interpersonal relationships, economic conditions, and institutional arrangements (Pawson and Tilley, 2004). In City and Hackney, the ‘buy-in’ from GPs within limited consultation time and the complexity of diagnosis given the range of complex co-morbidities appears to be an important factor influencing referrals to social prescribing. Increases in the number of referrals are likely to occur where a feedback mechanism is in place as a reminder of the availability of the service and to highlight patients’ positive experiences. At the stage of the SPC, SPCs skills mix and location (whether in the GP surgery or not), and the amount of time spent with the patient appear to be of crucial importance. Although there is no conclusive evidence as to whether face-to-face interaction is important more widely in health interventions, this has certainly been vital in City and Hackney and other social prescribing interventions (Brandling and House, 2007). At the stage of community organisations, location and availability of services was an important contextual factor. It is important to consider that Hackney has a wide range and number of third and voluntary sector organisations on offer which constitute an essential infrastructure for the development of social prescribing. Interviews with small- and medium-sized third and voluntary sector stakeholders also revealed that one of the most significant contextual factors is the lack of funding availability to deliver services. There appears to be some justification for this response as micro, small, and medium size voluntary organisations have experienced an overall decline of income from government (NCVS, 2016) whilst large organisations have seen a substantial increase in income from government.

## Conclusions

The initial hypothesis of this realist evaluation was that ‘a social prescribing intervention improves wellbeing outcomes for patients suffering from isolation and mild mental health problems by providing a support mechanism (GPs, social prescribing coordinators and community organisations) which enable each patient to consider a set of alternative actions and thus embark in changing or more effectively managing their current health’ (Table 2). Although it is too early to draw firm conclusions, we identified and discussed a range of contextual factors and mechanisms that can inform the work of stakeholders involved in the development of social prescribing models across the United Kingdom and beyond. We also discuss some methodological issues of realist evaluation and consider some ideas for future research on social prescribing.

A range of interventions linking primary care and the third sector have been developed in the United Kingdom and beyond over many years (Pavey *et al*., 2011). However, these have been characterised by little or no involvement of a SPC. Social prescribing in City and Hackney and in at least 30 other places in the United Kingdom (Polley *et al*., 2017) represents a more novel and ‘holistic’ (Kimberlee, 2015) approach to pathways linking primary care with the third sector. The key mechanism underpinning social prescribing in City and Hackney is the social interaction between the patient and other stakeholders at various stages of the pathway, first with the GP, then with the SPC and third sector organisations. In particular, the relationship between patient and SPC deserves further attention as it appears to translate into practice some of the theories underpinning social cognitive theory, self-efficacy (Coulter and Ellins, 2006), motivation (Hibbard and Gilburt, 2014) which have been discussed in the previous section.

Future research in this area should concentrate on testing these theories by measuring quantitatively the impact of the SPC on patients, in addition to measuring changes in patients’ health outcomes across the pathway as a whole. A suitable comparison group with no access to SPC should be identified, possibly through randomised selection. This exercise would finally provide us with greater understanding of the role of SPCs in patients’ health and well-being and enable us to test social cognitive theory and develop a more precise framework for patient activation (Hibbard and Gilburt, 2014).

Moreover, as the SPC’s role is complex and requires a mix of skills and attitudes outside clinical knowledge, it raises important questions about training needs for SPCs who can play an important link between primary care and community organisations, and crucially help to relieve pressure on GPs with patients who do not have a clearly diagnosed clinical problem.

SPCs are also present in fields beyond social prescribing (HEE, 2016; HSCAS, 2016) which suggests that more lessons could be learnt about their impact. In addition, resources from different fields could be joined up to make more effective use of their work and maximise their effectiveness and cost-effectiveness. This also offers an opportunity to explore the evidence on SPCs more broadly through a systematic review of their contribution in different fields and identify potential lessons to be learnt from their experience in different fields.

A wide range of contextual factors have been discussed in this article. GPs are an essential part of SP as they can use their reputation to convince patients to attend appointments with SPCs, and therefore overcome issues encountered among many such interventions, namely the lack of participation, particularly in the case of most vulnerable patients. Yet, most GPs have limited knowledge of third sector services’ availability, and when they have it, they find it difficult to keep up with the rate of activity turnover. The knowledge of community organisations and the links to the third sector as well as the knowledge of its ‘modus operandi’ was an important skill SPCs could deploy in assisting GPs. The number of referrals from GPs was assessed as lower than it should have been and particular efforts put in place to maximise referral rates including introducing ‘a pop up’ alert system on GP surgeries’ monitoring systems as well as, co-location of SPCs in GP surgeries. Attempts were also made to increase feedback mechanisms to highlight the development of the intervention. Lower than expected number of referrals was due to a range of reasons. First, the lack of a benchmark and little prior evaluation meant that initial expectations were overoptimistic; second, busy consultations provided GPs with little time to consider an alternative option for their patients; third, as GPs received extensive clinical training throughout their career, they would first consider a clinical rather than non-clinical route for their patients’ treatment.

In order to tackle most of these contextual issues time needs to be spent in building relationships between GPs and SPCs. At this stage, an important brokerage role can be played by Clinical Commissioning Groups. A similar point about the need to leave more time at the beginning of the process was raised in another realist evaluation of social prescribing (Arain, 2015).

Social prescribing has been introduced with different target groups in mind including people experiencing low and moderate mental health problems, long-term conditions such as diabetes, the socially isolated as well as, and people facing employment, benefit and/or housing issues. Whilst there is not yet clear evidence as to what target groups benefit the most, when delivered appropriately, social prescribing appears to motivate people to take action which could be applied to a range of different areas of health and social care. However, it is important to remember that another key contextual factor is the presence of a dynamic third sector which can accommodate and appropriately follow patients referred by SPCs. Many small organisations in City and Hackney support specific groups and have developed an important knowledge on how to reach and involve such groups. Whilst government investment supports many extremely large third sector organisations, small organisations face an overall reduction in their income from government (NCVS, 2016). Thus, greater investment in such organisations seems to be important for the long-term sustainability of social prescribing.

In addition to these conceptual and policy considerations, this article also raises an important methodological discussion about realist evaluation. In particular, we found that the explanatory power of realist evaluation is stronger when the intervention is sub-divided into different CMO configurations as this enables us to unpack different contextual factors and mechanisms operating at different stages. However, as argued by Jagosh *et al*. (2015), more needs to be done in relating the outcome of each stage with the context for a further stage of the CMO configuration. Moreover, another point of reflection for realist evaluation is that mechanisms cannot be triggered as an on/off switch as argued by Pawson and Tilley (2004) in their initial description of mechanism. The mechanisms operating in social prescribing are based on increasing trust between the patient and other stakeholders. As trust takes time to develop, a conclusion from this realist evaluation is that mechanisms should be conceived as a spectrum from a low to high level.

Finally, we wish to point out some of the limitations of this realist evaluation. It would be inappropriate to assume that the discussion in this realist evaluation applies to all social prescribing pilots as there are currently many different models of social prescribing. Some of these models include a stronger role for the SPC than others (Kimberlee, 2015), some focus more on prevention than treatment (eg, Ways to Wellness), and some have different sources of referrals beyond GP practices (eg, Waltham Forest social prescribing service). However, the mechanisms and contextual factors discussed in this article could inform discussion in other models and other areas. For instance, the relationship between SPC and patient and the role of the SPC are key to the appropriate functioning of the social prescribing pathway. Furthermore, on a more methodological point, the realist evaluation would have benefitted from more information about the type and frequency of patients’ attendance to community activities to understand more about for whom and under what circumstances this intervention is likely to work. This evaluation would have also benefitted from more interviews with GPs in order to capture the variety of views within primary care (Popay *et al*., 2007).

Realist evaluation gave us additional insights into the social prescribing initiative above and beyond the ‘effectiveness’ outcomes we collected through more conventional evaluation methodologies (Bertotti *et al*., 2015; Carnes *et al*., 2015). It offered a range of useful lessons to be considered, particularly at the design stage. Referrals from GP practices can vary considerably, thus SPCs need to invest time in forging and maintaining relationships with GP practices. Furthermore, commissioners of social prescribing services should consider carefully the role of the SPC. It is important to select and train SPCs carefully. SPCs need to be professional, empathetic and motivated by their contribution to both patients and the communities they support. Finally, funding and training for the community and voluntary sector is critical for the success of any social prescribing service. Yet, only few examples of social prescribing such as Rotherham (Dayson *et al*., 2013) seem to have invested in building capacity and sustaining this sector.

**Table 2:** Logic model for the realist evaluation of social prescribing in city and Hackney (London)